# Synthesis and pharmacokinetic characterisation of a fluorine-18 labelled brain shuttle peptide fusion dimeric affibody

**DOI:** 10.1038/s41598-021-82037-2

**Published:** 2021-01-28

**Authors:** Takahiro Morito, Ryuichi Harada, Ren Iwata, Yiqing Du, Nobuyuki Okamura, Yukitsuka Kudo, Kazuhiko Yanai

**Affiliations:** 1 Department of Pharmacology, Tohoku University Graduate School of Medicine, Seiryo-machi 2-1, Aoba-ku, Sendai, Miyagi 9808575 Japan; 2 Division of Brain Science, Department of Geriatrics and Gerontology, Institute of Development, Aging and Cancer, Tohoku University, Sendai, Miyagi Japan; 3 Cyclotron and Radioisotope Center, Tohoku University, Sendai, Miyagi Japan; 4 Division of Pharmacology, Faculty of Medicine, Tohoku Medical and Pharmaceutical University, Sendai, Miyagi Japan

**Keywords:** Imaging, Positron-emission tomography, Protein delivery, Pharmacokinetics

## Abstract

Brain positron emission tomography (PET) imaging with radiolabelled proteins is an emerging concept that potentially enables visualization of unique molecular targets in the brain. However, the pharmacokinetics and protein radiolabelling methods remain challenging. Here, we report the performance of an engineered, blood–brain barrier (BBB)-permeable affibody molecule that exhibits rapid clearance from the brain, which was radiolabelled using a unique fluorine-18 labelling method, a cell-free protein radiosynthesis (CFPRS) system. AS69, a small (14 kDa) dimeric affibody molecule that binds to the monomeric and oligomeric states of α-synuclein, was newly designed for brain delivery with an apolipoprotein E (ApoE)-derived brain shuttle peptide as AS69-ApoE (22 kDa). The radiolabelled products ^18^F-AS69 and ^18^F-AS69-ApoE were successfully synthesised using the CFPRS system. Notably, ^18^F-AS69-ApoE showed higher BBB permeability than ^18^F-AS69 in an ex vivo study at 10 and 30 min post injection and was partially cleared from the brain at 120 min post injection. These results suggest that small, a brain shuttle peptide-fused fluorine-18 labelled protein binders can potentially be utilised for brain molecular imaging.

## Introduction

Positron emission tomography (PET) neuroimaging has been developed and utilised for clinical diagnosis, pathophysiological analysis, and drug development for neurodegenerative disorders^1–5^. Following the advent of the amyloid imaging concept with [^11^C]Pittsburgh compound B, blood–brain barrier (BBB)-permeable small compounds that recognise the cross β-sheet structure of amyloid fibrils have been exploited to visualise senile plaques or neurofibrillary tangles in Alzheimer’s disease (AD). Although several PET tracers have been approved by the Food and Drug Administration^6^, there remain challenges in the development of small molecule PET tracers for other misfolded proteins or non-amyloid conformations with their difficulty in producing with a high target selectivity and affinity^7^. A typical example is α-synuclein imaging; it has also been troubled by the low selectivity and affinity of candidate compounds^8^.

Recently, a research group demonstrated proof-of-concept for neuroimaging with antibody-based protein binders in AD model mice^9–12^. Since the proteins can be designed using protein engineering technologies to achieve high selectivity and affinity against specific molecules, this strategy potentially expands the spectrum of existing molecular imaging targets and can provide an option for brain imaging strategies. However, there are challenges in the pharmacokinetics and radiolabelling methods for these proteins^13^. First, antibody-based protein binders exhibit a long biological half-life (> 3 h) in the blood, taking a long time to acquire high-contrast images, which brings a need to use PET radionuclides with a longer half-life (e.g., iodine-124, with a half-life of 100.2 h). Second, the direct ionisation method for radiolabelling of reported proteins with iodine-124 is not a universal method because it can hinder the proper functioning of these proteins via redox reactions with chloramine-T and non-site-specific ionisation of tyrosine. Ideally, radiotracers should show rapid clearance from the body and can easily be radiolabelled with radionuclides with a shorter half-life, such as fluorine-18, for clinical practicability.

Affibody molecules, which are small (approximately 7 kDa) protein ligands with a high affinity and selectivity to specific targets, have been recently identified through protein engineering using phage or yeast display^14–16^. They possess favourable pharmacokinetics, enabling the acquisition of high-contrast images in several hours if they were radiolabelled with positron emitters with short half-lives such as fluorine-18, eliciting interest in their application as PET imaging agents^16,17^. Recently, the dimeric affibody AS69 (14 kDa) was reported as a ligand of α-synuclein, binding to monomers (*K*_D_ = 250 nM) and oligomers (*K*_D_ = 32.3 nM), but not to amyloid-β (Aβ) monomers (*K*_D_ = 5000 nM)^18–20^. The major challenge of small molecules for α-synuclein imaging is their low selectivity over other misfolded proteins such as Aβ. Thus, AS69 can be considered as a potential candidate for an α-synuclein PET tracer with its target selectivity; however, it was expected to show poor penetration of the blood–brain barrier like general protein molecules. That would be an impediment for neuroimaging applications.

**Figure 1:**
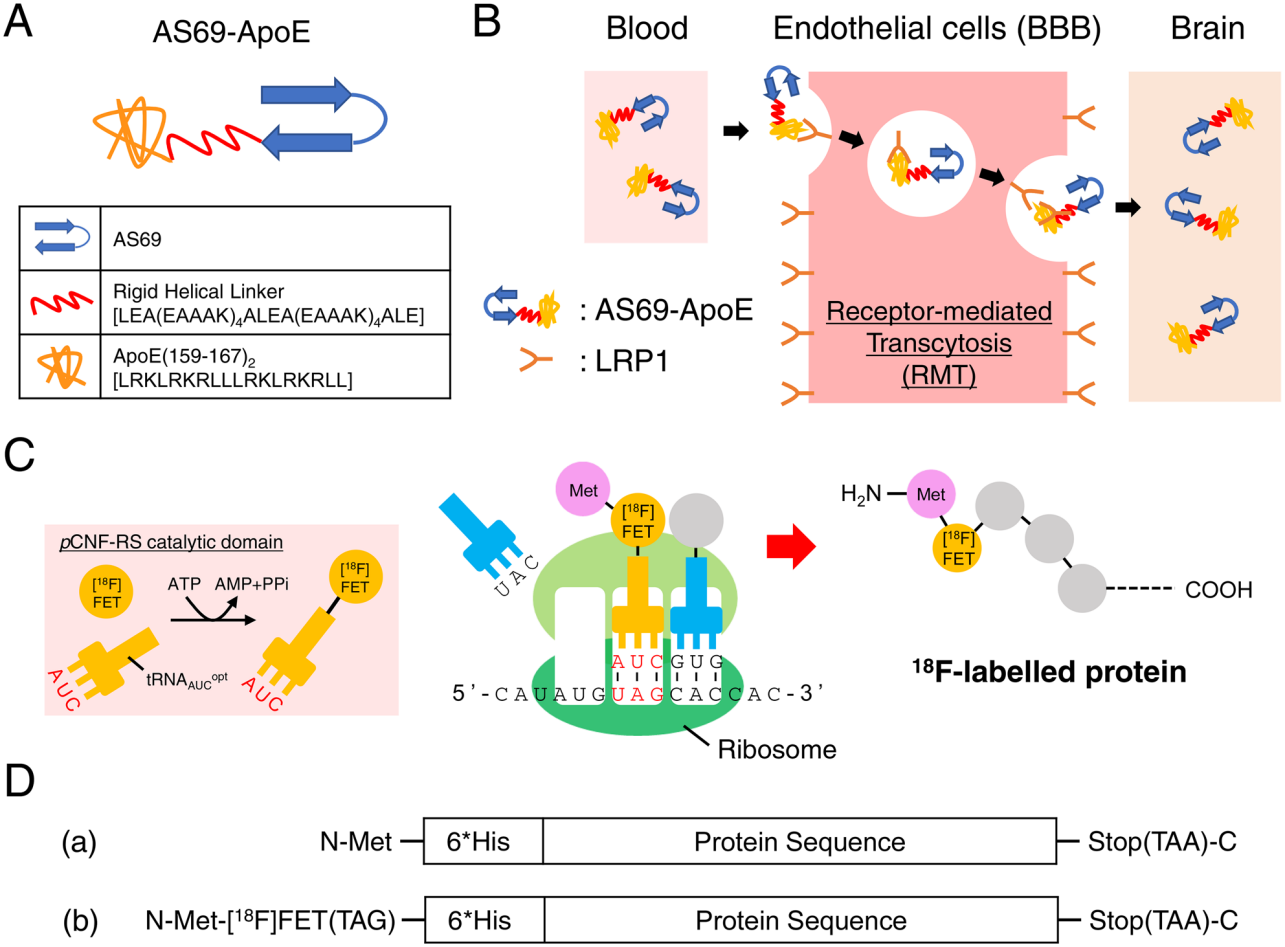
Schematic description of the study design and strategy. **(A)** Molecular design of AS69-ApoE. AS69 and ApoE (159–167)_2_ peptides were genetically linked by a rigid helical linker. The linker sequence and ApoE (159–167)_2_ peptide sequence are described in brackets. **(B)** Schematic diagram of receptor-mediated transcytosis (RMT) induced by the ApoE (159–167)_2_ peptide via low-density lipoprotein receptor-related protein 1 (LRP1). ApoE (159–167)_2_ peptide recognises LRP1 expressed on blood endothelial cells, and the fusion protein is transported into the brain via RMT. **(C)** Schematic diagram of [^18^F]FET incorporation by genetic expansion in the cell-free protein radiosynthesis system^30^. *p*CNF-RS = *p*-cyanophenylalanine aminoacyl-tRNA synthetase; ATP = adenosine triphosphate; AMP = adenosine monophosphate; PPi = pyrophosphoric acid. **(D)** The design of (a) non-radiolabelled and (b) radiolabelled protein in this study. 6*His = polyhistidine-tag. These sequences were inserted into a pET-21a plasmid vector for bacterial expression (a) or cell-free protein radiosynthesis (b).

To overcome this issue, we utilised a brain shuttle peptide that enables cargo proteins to permeate the BBB via the receptor-mediated transcytosis (RMT) pathway^21,22^. The use of brain shuttle peptides is beneficial because of their small size (typically < 10 kDa) that enables smaller protein design, in contrast with the well-established strategy that also uses RMT for brain delivery with anti-transferrin receptor 1 antibody derivatives (> 28 kDa)^13,23,24^. We chose the apolipoprotein E (ApoE) (159–167)_2_ peptide, a brain shuttle peptide (2.4 kDa) composed of a tandem repeat of a partial ApoE sequence. The fusion of this peptide with α-L-iduronidase and arylsulfatase A (ASA) increased its cellular or mouse brain uptake by RMT via low-density lipoprotein receptor-related protein 1 (LRP1)^25,26^. The accumulation of ApoE peptide-fused ASA (referred to as ASA-ApoE-II in the literature) in mice brain was approximately 1.5- fold higher than that of ASA, at 120 min following intravenous injection. By genetic fusion of this ApoE (159–167)_2_ peptide to AS69 with a rigid helical linker, we designed AS69-ApoE (Fig. 1A, 22 kDa) with the aim of transporting the AS69 functional domain to the brain (Fig. 1B).The rigid helical linker reportedly increases protein stability and rigidly separates the two functional domains^27–29^.

Although several methods for protein radiolabelling with fluorine-18 have been reported, the development of mild, site-specific, and robust radiolabelling methods has been challenging^13,16^. As an option to solve these issues, we have developed a cell-free protein radiosynthesis (CFPRS) system for protein fluorine-18 labelling with a genetic expansion strategy using *p*-cyanophenylalanine aminoacyl-tRNA synthetase (*p*CNF-RS), tRNA optimised for amber codon recoding (tRNA_CUA_^opt^), and *O*-2-[^18^F]fluoroethyl-L-tyrosine ([^18^F]FET). The CFPRS system enables site-specific fluorine-18 radiolabelling of various proteins with a high molar activity under mild conditions and with short reaction times through a simple addition of the template DNA^30^. In the biological translation process of the CFPRS system, the amber codon inserted after the start codon of the original sequence encodes the [^18^F]FET, resulting in the facile radiolabelling of proteins (Fig. 1C,D). Using this CFPRS system, we successfully labelled an affibody molecule that binds to human epidermal growth factor receptor type 2 with fluorine-18 and demonstrated the utility of the construct in tumour PET imaging^30^.

In the present study, we determined the binding characteristics of AS69 and the newly designed AS69-ApoE construct in vitro and radiolabelled AS69 and AS69-ApoE with fluorine-18 (obtaining ^18^F-AS69 and ^18^F-AS69-ApoE) using the CFPRS system. We used ^18^F-AS69 and ^18^F-AS69-ApoE in wild-type mice to evaluate and compare their pharmacokinetics and to assess the brain penetration of the labelled proteins.

## Results

### In vitro characterisation of AS69 and AS69-ApoE

**Figure 2:**
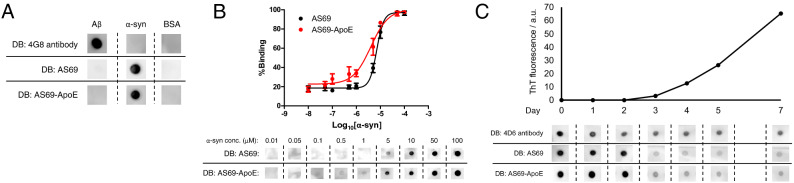
Binding properties of AS69 and AS69-ApoE evaluated by dot blot. **(A)** Binding affinity of AS69 and AS69-ApoE against amyloid-β 1–42 (Aβ_1-42_) and α-synuclein monomers. Aβ_1-42_ and α-synuclein monomers were blotted on a nitrocellulose membrane at a concentration of 100 µM. AS69 and AS69-ApoE were detected by anti-His-tag antibody. Bovine serum albumin (BSA) was used as a negative control. **(B)** Binding of AS69 and AS69-ApoE with different concentrations of α-synuclein monomers (10 nM–100 µM) (n = 5). The percentage of binding was calculated from grey values. **(C)** Binding characteristics against different conformations of α-synuclein. The α-synuclein monomers (280 µM) were incubated and sampled after 0, 1, 2, 3, 4, 5, and 7 days. The thioflavin T fluorescence assay was performed on each sample to monitor fibrilisation status. a.u., arbitrary unit.

First, we investigated the binding selectivity of AS69 and AS69-ApoE against α-synuclein and Aβ_1-42_ monomers through dot blot (Fig. 2A). Both AS69 and AS69-ApoE showed selective binding to α-synuclein monomers but not to Aβ_1-42_ monomers, suggesting that AS69-ApoE retained the binding properties of AS69. Subsequently, we evaluated the binding affinity of AS69 and AS69-ApoE against α-synuclein monomers (Fig. 2B). AS69-ApoE retained the binding affinity of AS69; however, its binding affinity was slightly improved. The EC_50_ values were 7.05 µM for AS69 (95% CI 6.25–7.95 µM) and 4.01 µM for AS69-ApoE (95% CI 2.58–6.22 µM), which indicated the effect of ApoE peptide fusion to AS69 on its binding forms. Furthermore, we evaluated the binding properties of AS69 and AS69-ApoE against different conformations of α-synuclein (Fig. 2C). The morphological state of α-synuclein was monitored via ThT fluorescence, which reflected the amount of fibril structure present^31^. The signals of AS69 and AS69-ApoE binding declined in a time-dependent manner, suggesting that AS69 and AS69-ApoE bound to ThT-negative α-synuclein conformations but showed less binding to ThT-positive aggregated α-synuclein.

### Radiosynthesis of ^18^F-AS69 and ^18^F-AS69-ApoE

**Figure 3:**
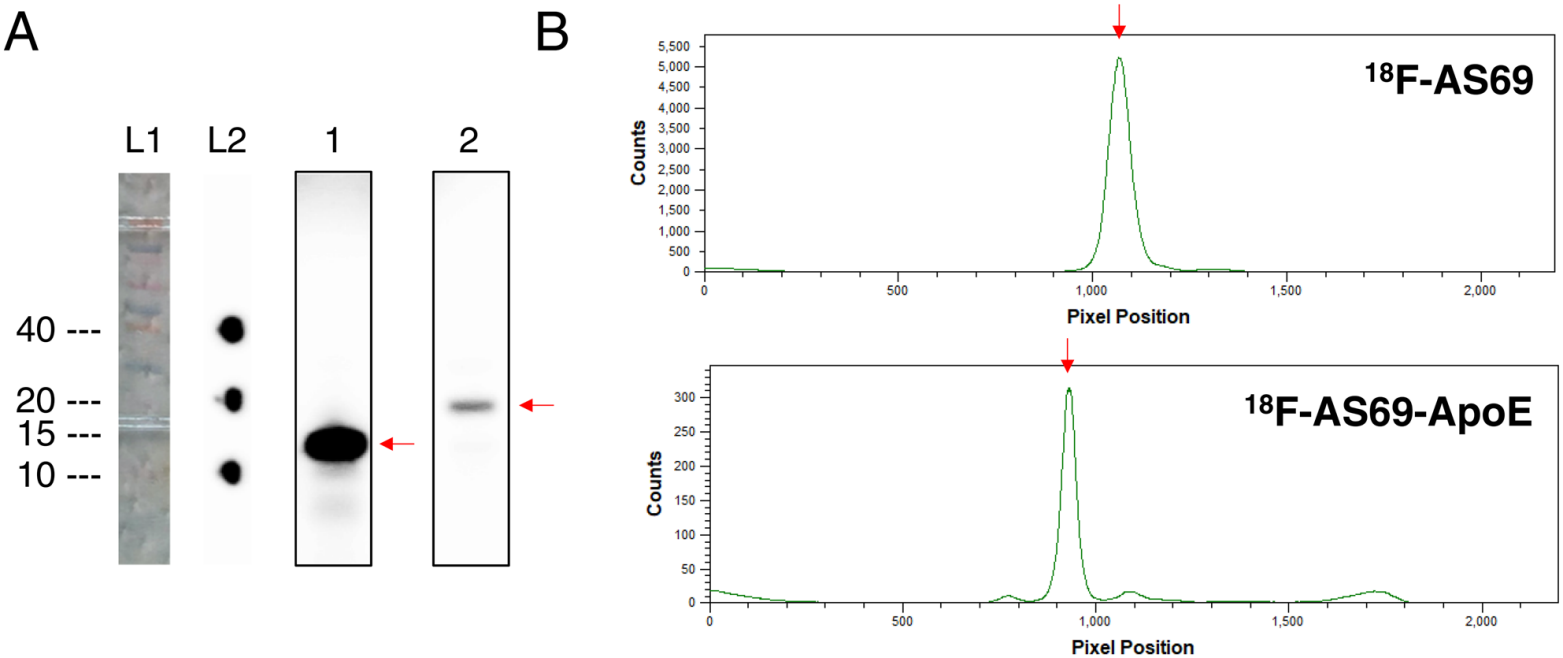
Radiochemical purities of ^18^F-AS69 and ^18^F-AS69-ApoE. **(A)** SDS-PAGE analysis of purified proteins. The molecular weights of ^18^F-AS69 and ^18^F-AS69-ApoE are 14 kDa and 22 kDa, respectively. Ladder 1 (L1): original ladder lane from a gel; Ladder 2 (L2): autoradiographic markers at the position of 10, 20, and 40 kDa ladder bands; Lane 1 and 2: the purified fractions of ^18^F-AS69 and ^18^F-AS69-ApoE, respectively. **(B)** Assessment of radiochemical purity via a gel image analysis from lane 1 and 2. Original autographic images and gels were shown in Fig. S1.

^18^F-AS69 and ^18^F-AS69-ApoE were successfully synthesised by the CFPRS system via the proposed mechanism described in Fig. 1C within 60 min, which included the purification steps. Analysis of purification integrity using SDS-PAGE is shown in Fig. 3A and Fig S1, which shows a single band in lane 1 and 2 derived from the purified, radioactive proteins, indicating the successful preparation of ^18^F-AS69 and ^18^F-AS69-ApoE. The radiochemical purity (radioactivity from main products/radioactivity from total products) of each construct was estimated as > 95% by image analysis (Fig. 3B), and the decay-corrected radiochemical yields (RCY, radioactivity from main products/total radioactivity used) of ^18^F-AS69 and ^18^F-AS69-ApoE were 17.0% ± 1.98% and 2.79% ± 0.84%, immediately following His-tag purification. The estimated molar activity of the radiolabelled proteins was 498 ± 151 GBq/μmol after a 30-min reaction, based on the molar activity of [^18^F]FET at the reaction start time (549 ± 174 GBq/μmol). The molar activity of each experiment is shown in Table S1.

### Biodistribution study of ^18^F-AS69 and ^18^F-AS69-ApoE

**Figure 4:**
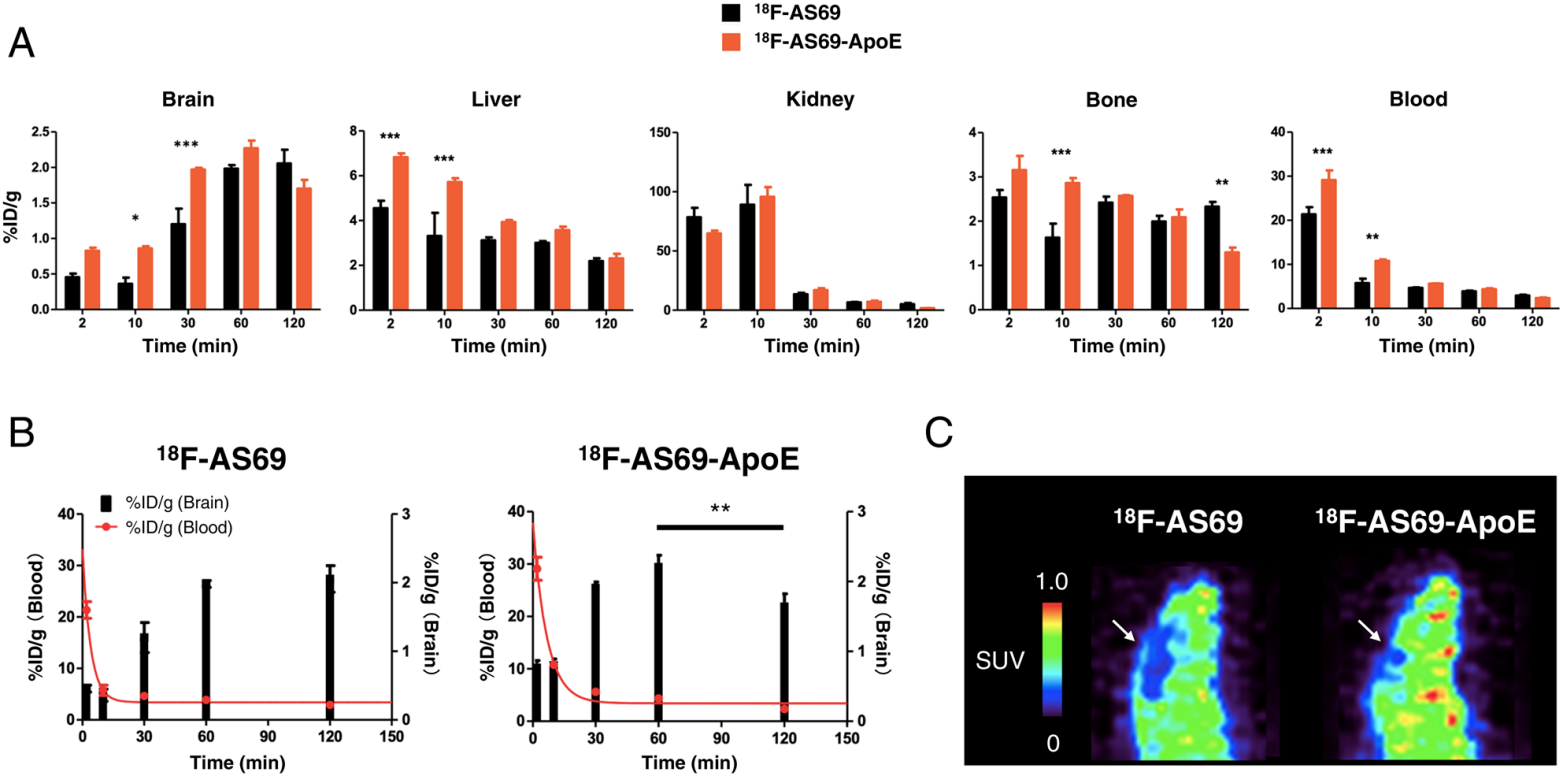
Ex vivo and PET biodistribution study in mice using ^18^F-AS69 and ^18^F-AS69-ApoE. **(A)** Normalised radioactivity [percentage of injected dose/g (%ID/g)] from the mouse brain at each time point [^18^F-AS69-ApoE: 2 and 10 min (n = 3); ^18^F-AS69: 10, 30 and 120 min; ^18^F-AS69-ApoE: 30 min (n = 4); ^18^F-AS69-ApoE: 120 min (n = 5); ^18^F-AS69: 2 and 60 min; ^18^F-AS69-ApoE: 60 min (n = 7)]. The black and orange bars indicate the %ID/g value ± SEM in mice administered ^18^F-AS69 and ^18^F-AS69-ApoE, respectively. The injected dose in all experiments ranged from 185–370 kBq. Statistical significance was calculated using two-way ANOVA and Sidak’s multiple comparisons test (*p < 0.05, **p < 0.01, and ***p < 0.001). **(B)** Comparison of time–activity curves in the brain and blood following the administration of ^18^F-AS69 and ^18^F-AS69-ApoE. The values of %ID/g in the blood (red dot) were fitted to a one-phase decay model, and the curve (red) was drawn. Black bars indicate the %ID/g value ± SEM. Statistical analysis was performed using two-way ANOVA and Sidak’s multiple comparisons test (**p < 0.01). **(C)** PET images with ^18^F-AS69 and ^18^F-AS69-ApoE in the mouse brain (25–30 min post-injection, sagittal views). White arrows indicate the position of the mouse brain.

Next, we compared the ex vivo biodistribution of ^18^F-AS69 and ^18^F-AS69-ApoE in wild-type mice (slc:ICR, male; Fig. 4A). The detailed values are shown in Table S2. The brain accumulation of ^18^F-AS69-ApoE was significantly higher than that of ^18^F-AS69 at 10 min and 30 min post-injection (*p* = 0.045 and *p* = 0.0003, respectively). The ratios of %ID/g for the brain (^18^F-AS69/^18^F-AS69-ApoE) at each time point were 2.38 and 1.64, respectively. In addition, ^18^F-AS69 was retained in the brain at 120 min post-injection; however, ^18^F-AS69-ApoE levels were significantly reduced (Fig. 4B, p = 0.0022). The estimated clearance rate of ^18^F-AS69-ApoE from 60 to 120 min was 0.013 (%ID/g)/min (the difference between the %ID/g values at the 60 and 120 min/60 min), which indicates faster clearance of ^18^F-AS69-ApoE from the brain. The tracer accumulation in the liver increased at the early time points, as predicted in the literature^26^. The blood concentration curve and tracer accumulation in the brain are presented in Fig. 4B and show that both ^18^F-AS69 and ^18^F-AS69-ApoE were rapidly cleared from the blood circulation within 10 min. The fitted biological half-lives of ^18^F-AS69 and ^18^F-AS69-ApoE were 2.67 min (95% CI 1.76–6.13 min) and 4.41 min (95% CI 3.29–6.71 min), respectively. Additionally, PET imaging was performed using ^18^F-AS69 and ^18^F-AS69-ApoE in wild-type mice (Fig. 4C). The images from the mice at 25–30 min after the injection of ^18^F-AS69-ApoE showed increased SUV values in the brain compared with those from the ^18^F-AS69-administered mice, which was consistent with the results from the ex vivo biodistribution study. PET images of other timeframes are shown in Fig S2.

### In vitro stability of ^18^F-AS69 and ^18^F-AS69-ApoE

**Figure 5:**
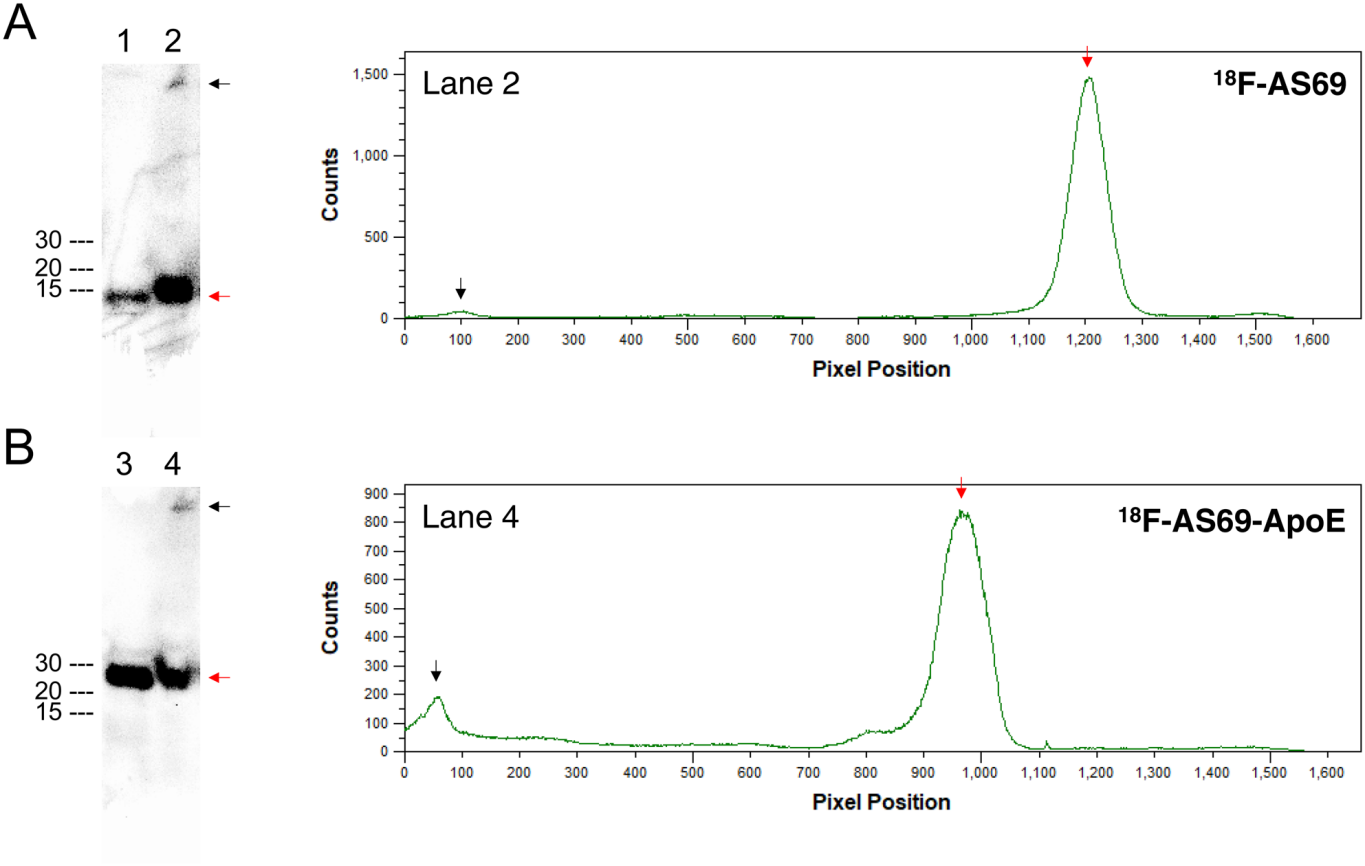
In vitro stability of ^18^F-AS69 and ^18^F-AS69-ApoE in mouse plasma. Results for ^18^F-AS69 (A, 14 kDa) and ^18^F-AS69-ApoE (B, 22 kDa). The figure presents autoradiographs from gels following SDS-PAGE analysis of ^18^F-AS69 and ^18^F-AS69-ApoE samples incubated for 60 min at 37 °C in PBS (lane 1 and 3) or mouse plasma (lane 2 and 4), and image analysis of lanes 2 and 4. Red arrows indicate the major products (^18^F-AS69 in A, ^18^F-AS69-ApoE in B) and black arrows indicate the radioactivity appeared at the top of each lane. The radioactivity of the samples was 18.5–37.0 kBq/10 µL when they were applied. Full-length autographic images and gels were shown in Fig. S3.

To support the idea that ^18^F-AS69-ApoE, not its metabolites, crossed the BBB and showed radioactivity in the brain, we evaluated the stability of ^18^F-AS69 and ^18^F-AS69-ApoE in mouse plasma in vitro. Following a 60-min incubation of ^18^F-AS69 and ^18^F-AS69-ApoE in the mouse plasma, most radioactivity remained detectable in intact forms (Fig. 5A,B), indicating that both radiolabelled proteins were mostly stable in the mouse plasma as well as the 10-, 30-, and 60-min incubation results (Fig. S3). On the other hand, some weak signals were also observed at the top of the gels.

### In vivo stability of ^18^F-AS69 and ^18^F-AS69-ApoE

**Figure 6:**
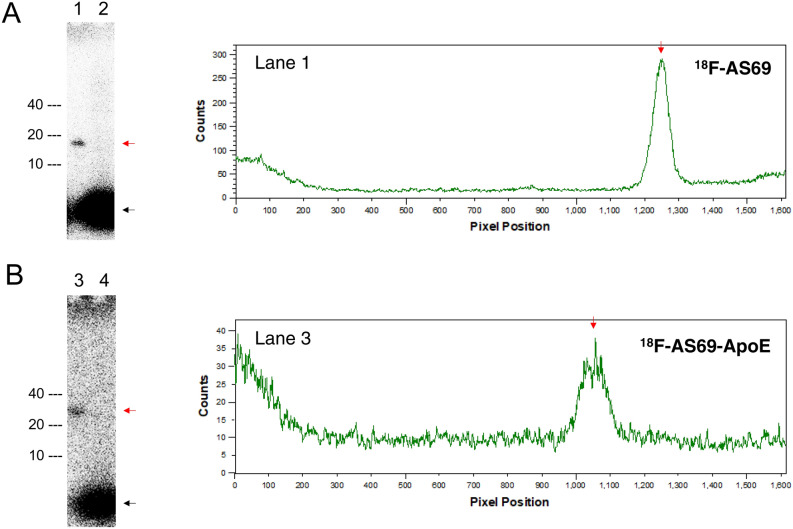
In vivo stability of ^18^F-AS69 and ^18^F-AS69-ApoE in mice. Results for ^18^F-AS69 (A, 14 kDa) and ^18^F-AS69-ApoE (B, 22 kDa). The figure presents autoradiographs from gels following SDS-PAGE analysis of mouse urine or blood samples collected as described in the experimental section. Blood (lane 1 and 3) or urine (lane 2 and 4) samples of mice collected 30 min after injection of ^18^F-AS69 (lane 1 and 2) or ^18^F-AS69-ApoE (lane 3 and 4) were tested. Black arrows indicate signals from mouse urine samples. Full-length autographic images and gels were shown in Fig. S4.

To confirm the in vivo stability of ^18^F-AS69-ApoE, plasma and urine samples were analysed via SDS-PAGE autoradiography at 30 min after intravenous administration of ^18^F-AS69 or ^18^F-AS69-ApoE into mice (Fig. 6A,B, Fig S4). We found that both ^18^F-AS69 and ^18^F-AS69-ApoE were predominantly detected in the plasma, suggesting that protein tracers can exist as their intact forms in the body. Furthermore, we also investigated excreted mouse urine, because the ex vivo results (Fig. 4A) and PET images (Fig. S2) showed quick accumulation in the kidney. No parent radiolabelled proteins were observed in the urine, while the dominant unknown radioactivity at the bottom of the gel was observed. It was not [^18^F]FET because it did not migrate on the NuPAGE gel as described in Fig. S1. This result indicates that ^18^F-AS69 and ^18^F-AS69-ApoE were completely metabolised or degraded before urinary excretion.

## Discussion

While brain PET imaging of senile plaques and neurofibrillary tangles with ^18^F-labelled small molecules has been successfully utilised for the diagnosis and drug development of AD, there remain challenges to visualise other misfolded proteins found in neurodegenerative diseases. Typical small molecule PET tracers recognise the rigid β-sheet structure of amyloid fibrils; however, this can be a limitation for visualising other misfolded proteins (e.g., α-synuclein and TAR DNA-binding protein 43). Hence, alternative strategies using BBB-permeable protein radiotracers have the potential to overcome limitations of the conventional approach with their unique binding mechanisms and would provide new insights into the field of neuroscience and neuroimaging.

In this study, we investigated the possibility of brain PET imaging with fluorine-18-labelled proteins as the first attempt. Fluorine-18 is the most widely used radionuclide because its decay half-life (t_1/2_ = 109.8 min) is appropriate for clinical applications^16^. On the other hand, we needed to design a protein molecule that reaches the brain and is cleared within the optimal time window of fluorine-18 (approximately 2 h). For this purpose, we focussed on affibody molecules that would be quickly eliminated from the bloodstream, and AS69 was selected as a potential candidate protein as an α-synuclein imaging tracer. We then designed a BBB-permeable affibody molecule, AS69-ApoE, by fusing the ApoE (159–167)_2_ peptide (Fig. 1A). This molecular design did not affect the binding properties of AS69, as previously reported^27,28^, suggesting that this strategy might be applicable to other proteins. Furthermore, we found that AS69-ApoE exhibited a slightly stronger binding affinity against α-synuclein monomers than AS69, while maintaining binding selectivity (Fig. 2B). This may be due to the electrostatic interactions between the positively charged ApoE (159–167)_2_ peptide (pI 12.70) and the negatively charged α-synuclein (pI 4.67). Our binding assay additionally showed that both AS69 and AS69-ApoE exhibited poor binding against α-synuclein samples when ThT fluorescence was detected (Fig. 2C), suggesting that AS69 and AS69-ApoE have low affinity against α-synuclein fibrils and limited utility for the visualisation of abnormal α-synuclein fibril accumulation in diseases^32^.

The production of ^18^F-AS69 and ^18^F-AS69-ApoE using the CFPRS system was successfully confirmed via gel autoradiography (Fig. 3A). However, the RCY remained relatively low compared with that obtained using other methods in previous studies^30^, suggesting that further system optimisation is required to improve RCY. Furthermore, the RCY of ^18^F-AS69-ApoE (2.79%) was less than that of ^18^F-AS69 (17.0%), probably because of the difference between AS69 and AS69-ApoE in terms of certain biological factors (e.g., translation efficacy and stability of product proteins). This indicates that the efficiency of the CFPRS system depends on the protein of interest. Although we could not directly measure the molar activity of ^18^F-AS69 and ^18^F-AS69-ApoE, this value can be estimated from the molar activity of [^18^F]FET because [^18^F]FET is theoretically incorporated in the protein in a 1:1 stoichiometry. Hence, we expected to synthesise both ^18^F-AS69 and ^18^F-AS69-ApoE with high molar activity comparable to that of [^18^F]FET (454 ± 144 GBq/μmol, immediately following 30 min of synthesis). This molar activity was remarkably higher than that from other methods of protein fluorine-18 labelling using N-succinimidyl 4-[^18^F]fluorobenzoate or aluminium ^18^F-fluoride-1,4,7-triazanonane-1,4,7-triacetate conjugate (typically 1–100 GBq/mol)^33–36^. This advantage allows for more sensitive target detection and high-contrast images. On the other hand, we cannot demonstrate in vitro binding experiments with both ^18^F-AS69 and ^18^F-AS69-ApoE. Based on the estimated molar activity, we used 50 nM of radiolabelled products, but this concentration was not sufficient to evaluate their binding against α-synuclein monomers (*K*_D_ = 250 nM). For further investigations, we need a protein binder that possess low nanomolar affinity.

The BBB permeability of AS69 was increased by the addition of a partial peptide of ApoE (Fig. 4A,C), which was consistent with previous findings^25,26^. We confirmed more than 2%ID/g brain uptake of ^18^F-AS69-ApoE at 30 min and 60 min post-injection. This value seems low compared with successful small radioligands for neuroimaging that showed over 4%ID/g in the brain within a few minutes of intravenous injection^7^. However, the PET images (Fig. S1) and ex vivo results (Fig. 4B) indicate that ^18^F-AS69-ApoE gradually entered the brain, in contrast with the dynamic pharmacokinetics of small compounds. Hence, small protein tracers have unique pharmacokinetic characteristics and may be difficult to apply to the principles of small-molecule PET tracers. In support of this consideration, amyloid brain imaging with antibody-based protein binders has been achieved with < 0.5%ID/g brain radioactivity^10,13^. Furthermore, we can exclude the effect of the blood activity on the brain radioactivity because a higher brain uptake was observed in ^18^F-AS69-ApoE despite having no significant difference between ^18^F-AS69 and ^18^F-AS69-ApoE in blood radioactivity at 30 min post-injection. Moreover, at earlier time points, blood activity was rapidly decreased from 2 to 10 min post-injection, but there was no obvious difference in brain uptake between 2 and 10 min post-injection, indicating that the effect of the blood radioactivity does not seem to be critical for the evaluation of radiotracer uptake into the brain. Additionally, the accumulation of AS69 in the liver was significantly increased at early time points in the biodistribution study, as predicted from the literature^26^, perhaps because of the cationic properties of the fused ApoE (159–167)_2_ peptide. Since cell-permeating peptides are typically positively charged^37^, the ApoE (159–167)_2_ peptide may also exhibit the propensity of cell-permeating peptides, accelerating accumulation in the liver.

Brain-targeting PET tracers must show rapid clearance from the brain to evaluate its retention at specific targets in the brain. ^18^F-AS69 was retained in the brain from 60 to 120 min post injection, while the amount of ^18^F-AS69-ApoE in the brain was reduced (Fig. 4B), suggesting that the clearance of ^18^F-AS69-ApoE from the brain was initiated between 60 and 120 min after administration. To our knowledge, this is the first report to demonstrate enhanced clearance from the brain following the addition of a brain shuttle peptide. One hypothesis is that ^18^F-AS69-ApoE in the brain tissue interacts with LRP1, which is expressed in the brain, and moves into the blood via the BBB according to its concentration (using the reverse pathway described in Fig. 1B), following the clearance of most of the ^18^F-AS69-ApoE in the blood. This hypothesis implies that the BBB permeability of ^18^F-AS69-ApoE is directly related to its clearance from the brain. This finding is based on the short biological half-lives of affibody molecules, unlike antibodies that exhibit longer biological half-lives. Hence, further optimisation of fused brain shuttle peptides will facilitate greater BBB permeation and subsequent rapid clearance.

For the quantitative measurement of target proteins in the brain, radiotracers must be stable, and their radioactive metabolites should not enter the brain^38^. Our biodistribution study showed no significant defluorination of the labelled proteins over 120 min following administration (Fig. 4A). However because both ^18^F-AS69 and ^18^F-AS69-ApoE were labelled with incorporated [^18^F]FET located adjacent to the first methionine, [^18^F]FET might be released from radiolabelled proteins by endogenous protease hydrolysis in vivo. [^18^F]FET was shown to cross the BBB probably via a specific amino acid transport system^39^; thus, [^18^F]FET potentially appears to be a representative radiolabelled metabolite that merits consideration. As presented in Fig. 5, the in vitro stability assay of ^18^F-AS69 and ^18^F-AS69-ApoE showed that they were stable in the mouse plasma for at least 60 min. The signals at the top of the gel might be [^18^F]FET that has the property to stay in the wells, or fluorine-18 labelled proteins that did not migrate into the gels by forming aggregates with other proteins. Notably, the in vivo stability assay demonstrated that both protein radiotracers are likely intact in mouse plasma at 30 min post-injection, supporting the idea that intact protein tracers entered the brain. However, no intact tracers were observed in mouse urine, indicating that protein tracers were thoroughly metabolised before urinary excretion. Most of the radioactivity in urine migrated into the gels was not [^18^F]FET with a reason above, but could be small molecular weight peptides or molecules. On the other hand, the limitation of this study is its inability to directly evaluate metabolites in the brain with two issues in analysing the radioactive fraction from the brain after intravenous administration of radiotracers. First, it was difficult to separate all radioactive protein fractions from whole brain tissues. Second, the radioactivity in the brain was relatively low compared to the blood, liver, and kidney (Fig. 4), to perform further analysis with SDS-PAGE autoradiography or column chromatography. Further metabolism analyses are required to fully characterise the in vivo stability of protein radiotracers.

Our goal in this study was to demonstrate in vivo brain PET imaging using mouse models and ^18^F-labelled proteins within several hours. However, there remain challenges that need to be addressed. First, to our knowledge, there are no protein binders available that meet the following requirements: (1) sufficiently small size to enable rapid clearance from the body, (2) ability to be produced in the CFPRS system, and (3) high affinity (*K*_*D*_ < 1 nM) and selectivity against target molecules in the brain. Second, although the ApoE (159–167)_2_ peptide fusion improved the brain penetration of AS69, we cannot conclude that the fusion of an ApoE shuttle peptide to protein binders is the most effective approach for visualisation of the brain within several hours. Hence, our future study will focus on the identification of protein binders suitable for protein brain PET imaging and the consideration of another shuttle peptide fusion.

In conclusion, a newly designed protein fused with a brain shuttle peptide, AS69-ApoE, retained the binding affinity and selectivity of AS69. We successfully labelled AS69 and AS69-ApoE with fluorine-18 using our CFPRS methods. The results additionally suggest that the CFPRS system may be applicable to general protein molecules. Notably, ^18^F-AS69-ApoE showed higher BBB permeability and faster clearance from the brain than ^18^F-AS69. This indicates that the strategy of shuttle peptide fusion to small proteins such as affibody molecules is potentially applicable for neuroimaging. Further optimisation of shuttle peptides and identification of small protein binders will enable molecular brain PET imaging with fluorine-18-labelled proteins.

## Materials and methods

### General

Restriction enzymes and RNase inhibitor (murine) were purchased from New England Biolabs (USA). Amyloid-β 1–42 (Aβ_1-42_, 0.55 mg) was purchased from the Peptide Institute (Japan). Protein concentration was determined using the Pierce BCA Protein Assay Kit (Thermo Fisher Scientific, USA). Proteins were concentrated via ultrafiltration using Amicon Ultra-15 filters (3000 nominal molecular weight limit, Merck Millipore, USA). Dot blotting was performed using a nitrocellulose membrane (Invitrogen, USA). Horseradish peroxidase (HRP)-conjugated secondary antibodies were purchased from Abcam (UK), HRP detection was performed with EzWestLumi plus (Atto, Japan), and tRNA_CUA_^opt^ was custom-synthesised by GeneDesign, Inc. (Japan).

### Preparation of α-synuclein, AS69, and AS69-ApoE

The pET-28a plasmid encoding human α-synuclein was a gift from Prof. Koji Sode of North Carolina State University (USA). The AS69 and AS69-ApoE genes were custom-synthesised (GeneScript, USA) and cloned into the expression vector pET-21a(+) into the *Nde*I/*Xho*I restriction site. Protein expression was induced by the addition of 0.5 mM isopropyl β-D-1-thiogalactopyranoside when the optical density of the bacterial solution at 600 nm reached 0.5–0.7. Subsequently, the cells were collected via centrifugation and lysed via sonication. From the crude solution, α-synuclein was purified by heating, followed by anion-change chromatography and size-exclusion chromatography (SEC) using HiTrap Q HP and HiLoad Superdex 75 16/60 preparatory-grade columns (Cytiva, USA), respectively. AS69 and AS69-ApoE were purified using immobilized metal ion-adsorption chromatography and SEC with HisTrap FF and HiLoad Superdex 75 16/60 prep grade columns, respectively. Its purity was confirmed by sodium dodecyl sulphate polyacrylamide gel electrophoresis (SDS-PAGE).

### In vitro binding characterisation of AS69 and AS69-ApoE

Monomeric Aβ_1-42_ was prepared by dissolving the peptides in 100% anhydrous dimethyl sulfoxide at 5 mM, followed by dilution of this solution to 100 µM with phosphate-buffered saline (PBS). Monomeric α-synuclein was prepared in PBS following gel filtration. BSA was used as a control. The samples were blotted on a cellulose membrane (1 µL/dot). The membrane was blocked with 10% skim milk in Tris-buffered saline containing 0.1% Tween 20 (TBST). To evaluate the difference in binding affinity between AS69 and AS69-ApoE, diluted α-synuclein samples in PBS (100 µM, 50 µM, 10 µM, 5 µM, 1 µM, 500 nM, 100 nM, 50 nM, and 10 nM) were blotted onto a cellulose membrane (1 µL each). For the monitoring of α-synuclein fibrillation, α-synuclein (140 µM) was incubated in PBS at 37 °C for 0–7 days with 10 mM sodium azide; sampling was performed every day. Aliquots of the collected samples (5 µL) were added to 95 µL of 25 µM thioflavin T (ThT), and fluorescence was measured using an FP-6300 spectrofluorometer (JASCO, Japan) using excitation at 440 nm and emission at 480 nm. The membranes were incubated for 60 min with 280 nM AS69 or AS69-ApoE for the assessment of their binding. Membrane-bound AS69 and AS69-ApoE were probed using anti-His-tag antibody (Proteintech, Japan, 1:1000 in TBST, incubated for 1 h at 20 °C) and HRP-conjugated secondary antibodies (1:1000 in TBST, incubated for 30 min at room temperature). Three rounds of washing for 5 min were performed between each procedure. Anti-Aβ antibody (4G8, 1:1000) and anti-α-synuclein antibody (4D6, 1:1000) were used as positive controls. HRP-driven chemical luminescence from the membrane was imaged with an Ez-Capture MG luminescent image analyser (ATTO). For evaluation of binding, grey values of detected dots on the membrane were measured using ImageJ software and analysed using GraphPad Prism 5 (GraphPad, USA).

### Radiosynthesis of [^18^F]FET

In this study, an unnatural, radiolabelled amino acid [^18^F]FET was used for protein radiolabelling using the CFPRS system because it is well characterised and optimised as a tracer for tumour imaging, and [^18^F]FET can be charged to tRNA_CUA_^opt^ by an engineered aminoacyl-tRNA synthetase (*p*CNF-RS). [^18^F]Fluoride was produced through the ^18^O(p,n)^18^F reaction on [^18^O]H_2_O (Taiyo-Nippon Sanso, Japan) with a Cypris HM-12 cyclotron (Sumitomo Heavy Industries, Japan) at the Cyclotron and Radioisotope Center of Tohoku University. [^18^F]FET was prepared by microscale radiosynthesis, as described previously^30,40^. Its molar activity was 549 ± 174 GBq/μmol at the beginning of protein radiosynthesis (n = 12). Dried [^18^F]FET was dissolved in the reconstitution buffer supplied with the RTS 100 *E. coli* HY Kit (Biotechrabbit GmbH, Germany) in order to load the maximum amount of [^18^F]FET into the system.

### Radiosynthesis of ^18^F-AS69 and ^18^F-AS69-ApoE

The CFPRS system was constructed using the RTS 100 *E. coli* HY Kit as previously described^30^. The additional components per 375 µL of the reaction mixture include *p*CNF-RS (300–375 µg), tRNA_CUA_^opt^ (50.3 µg), RNase inhibitor (300 U), template pET-21a plasmids (4.5 µg, amber codon inserted as described in Fig. 1D by site-directed mutagenesis), and [^18^F]FET. The prepared solution was allowed to react at 30 °C for 30 min. The synthesised proteins were purified using a His SpinTrap column (Cytiva) according to the manufacturer's instructions. For the experiments, the solvent was replaced with PBS using a NAP-5 column (Cytiva).

### Gel autoradiography

The radiochemical purity of the product was confirmed by SDS-PAGE based on the NuPAGE system (Invitrogen). To estimate the molecular weight of the products, Novex Sharp Pre-stained Protein Standard (Invitrogen) was loaded onto the first lane of each gel, and radioactive liquid was spotted on a fixed thin layer chromatography plate at the position corresponding to 10, 20, and 40 kDa standard bands. The gel was placed in contact with a BAS-IP TR 2025 imaging plate (Cytiva) overnight, and autoradiographic images were acquired using a Typhoon FLA 9500 laser scanner (Cytiva). The images were analysed using ImageQuant TL (Cytiva).

### Ex vivo biodistribution experiments in mice

All protocols using mice were approved by the Laboratory Animal Care Committee of Tohoku University and all animal experiments were performed in accordance with relevant guidelines and regulations including the ARRIVE guidelines. The mice (slc:ICR, male, 6–7 weeks) were anaesthetised, cervically dislocated, and dissected at defined time points (2, 10, 30, 60, and 120 min) after the intravenous injection of radiolabelled proteins (185–370 kBq/0.2 mL). The tissues (blood, brain, kidney, liver, and bone) were collected in vials (AS69-ApoE: 2 and 10 min (n = 3); AS69: 10, 30, and 120 min; AS69-ApoE: 30 min (n = 4); AS69-ApoE: 120 min (n = 5); AS69: 2 and 60 min; AS69-ApoE: 60 min (n = 7)]. The radioactivity of the respective tissues was measured using the gamma-ray counter AccFLEX γ7000 (Hitachi, Japan), and the counts were divided by the counts for injected radioactivity (decay-corrected) and the tissue weight for standardisation. The tissue weights were measured simultaneously using a gamma counter. In the figures, the standardised values are shown as the percentage of injected dose per gram (%ID/g). The values of %ID/g in the blood (red dot) were fitted to a one-phase decay model using GraphPad Prism 5. Results are shown as the mean ± standard error of the mean (SEM).

### Small-animal PET imaging

A PET study was performed using Clairvivo PET scanner (Shimadzu, Kyoto, Japan). Before the PET scans, the mice (slc:ICR, male, 6 w, n = 1) were anaesthetised with 1.5% (v/v) isoflurane. Emission scans were acquired for 120 min in three-dimensional (3D) list mode following intravenous administration of ^18^F-AS69 (4.56 MBq) or ^18^F-AS69-ApoE (3.34 MBq) dissolved in PBS via tail vein catheters. The resulting sinograms were reconstituted with the 3D-DRAMA algorithm into 25 frames (1 min × 5, 2 min × 5, 5 min × 9, and 10 min × 6). Standardised uptake value (SUV) images were obtained by normalising the tissue radioactivity concentrations according to the injected dose and body weight using the AMIDE software^41^.

### In vitro stability of protein tracers in mouse plasma

Mouse blood was collected via cardiac puncture using a syringe-connected needle previously wetted with heparin sodium (Mochida Pharmaceutical, Japan), followed by anaesthetisia of the mice and cervical dislocation for euthanasia, and centrifugation (1200×*g*) for 30 min at 4 °C. The cleared supernatants were carefully collected from the samples. The radiolabelled proteins (5 µL, 37–74 kBq) were added to 95 µL of PBS or mouse plasma and incubated for 60 min. The incubated samples (10 µL) were evaluated using gel-autoradiography as described above.

### In vivo stability of protein tracers in mice

^18^F-AS69 (2.96 MBq/0.2 mL) and ^18^F-AS69-ApoE (0.37 MBq/0.2 mL) were intravenously administered to mice [slc:ICR, male, 6w, ^18^F-AS69 (n = 3), ^18^F-AS69-ApoE (n = 2)]. Mice were anaesthetised with isoflurane and cervically dislocated 30 min post injection, then excreted urine and blood (> 100 μL) obtained from cardiac puncture were collected. The blood was centrifuged at 1200×*g* for 5 min, and the supernatant (10 μL) and the excreted urine (10 μL) were analysed by gel-autoradiography as described above.

### Statistical analysis

All statistical analyses were performed using GraphPad Prism 7. Significant differences were analysed with two-way ANOVA and Sidak’s multiple comparisons test (*p < 0.05, **p < 0.01, and ***p < 0.001) for comparison between the radiolabelled proteins with respect to their brain uptake and pharmacokinetics, respectively.

## Supplementary Information

Supplementary information.

## Data Availability

All data generated or analysed during this study are included in this published article (and its Supplementary Information files).
