# Supplementary material for: Synthesis and pharmacokinetic characterisation of a fluorine-18 labelled brain shuttle peptide fusion dimeric affibody
Source: Sci Rep. 2021 Jan 28;11:2588. doi: 10.1038/s41598-021-82037-2 (PMC7844286; doi:10.1038/s41598-021-82037-2)
Supplement: Supplementary file 1 — Supplementary information. [file 41598_2021_82037_MOESM1_ESM.docx]

Supplementary Information for

“Synthesis and Pharmacokinetic Characterisation of a Fluorine-18 Labelled Brain Shuttle Peptide Fusion Dimeric Affibody”

Takahiro Morito^1^, Ryuichi Harada^1,2*^, Ren Iwata^3^, Yiqing Du^1^, Nobuyuki Okamura^4^, Yukitsuka Kudo^2^ & Kazuhiko Yanai^1,3^

^1^Department of Pharmacology, Tohoku University Graduate School of Medicine, Sendai, Miyagi, JAPAN

^2^Department of Gerontology and Geriatrics, Division of Brain Science, Institute of Development, Aging and Cancer, Tohoku University, Sendai, Miyagi, JAPAN

^3^Cyclotron and Radioisotope Center, Tohoku University, Sendai, Miyagi, JAPAN

^4^Division of Pharmacology, Faculty of Medicine, Tohoku Medical and Pharmaceutical University, Sendai, Miyagi, JAPAN

*Corresponding author:

Ryuichi Harada, PhD

Assistant professor, Department of Pharmacology, Tohoku University School of Medicine, Seiryo-machi 2-1, Aoba-ku, Sendai 9808575, JAPAN

Tel: +81-22-717-8058, Fax: +81-22-717-8060

E-mail: [ryuichi.harada.c8@tohoku.ac.jp](mailto:ryuichi.harada.c8@tohoku.ac.jp)

Figure S1. The gel-autoradiography image and the original gel of Figure 3.

SDS-PAGE analysis of purified proteins. The molecular weights of ^18^F-AS69 and ^18^F-AS69-ApoE are 14 kDa and 22 kDa, respectively. Lane 1: crude solution (before reaction); Lane 2: flow-through; Lane 3: washing solution with PBS; lane 4: eluted solution with PBS, 500 mM imidazole. Black arrows indicate the position of [^18^F]FET and red arrows indicate the products (^18^F-AS69 and ^18^F-AS69-ApoE). Red dot squares indicate the cropped area used for Figure 3.


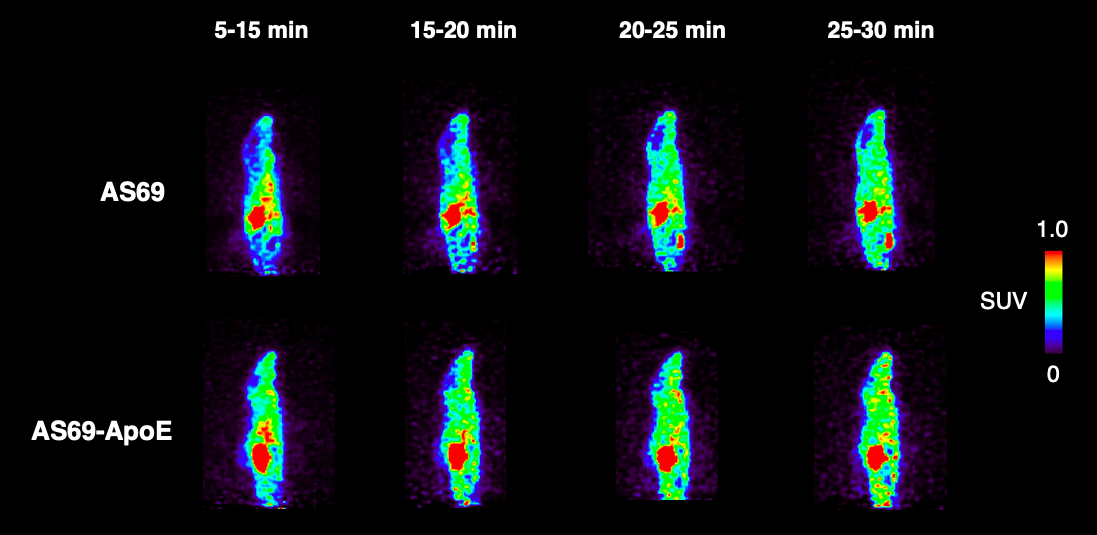


Figure S2. Sagittal PET Images at Other Timepoints.


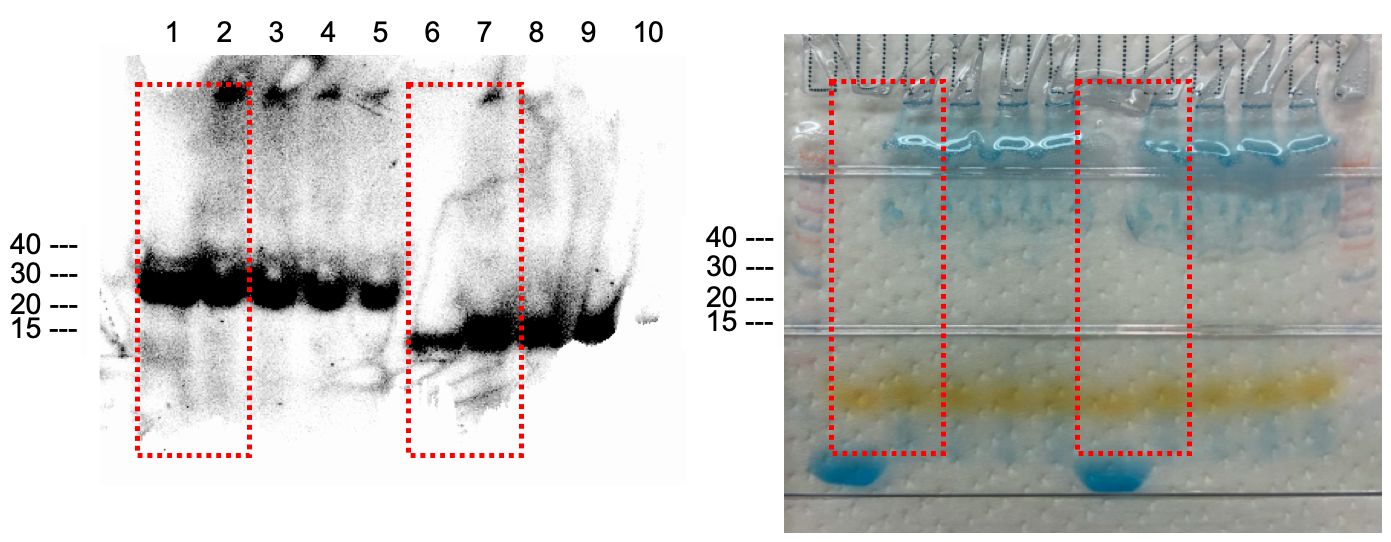


Figure S3. The gel-autoradiography image and the original gel of Figure 5.

^18^F-AS69-ApoE samples incubated in PBS for 60 min (lane 1) or in mouse plasma for 60, 30, 10, 0 min (lane 2-5) and ^18^F-AS69 samples incubated in PBS for 60 min (lane 6) or in mouse plasma for 60, 30, 10, 0 min (lane 7-10) were analysed by Gel-autoradiography. Lane 1-2 and lane 6-7 were respectively used for Figure 5A, B analysis. Red dot squares indicate the cropped area used for Figure 5.


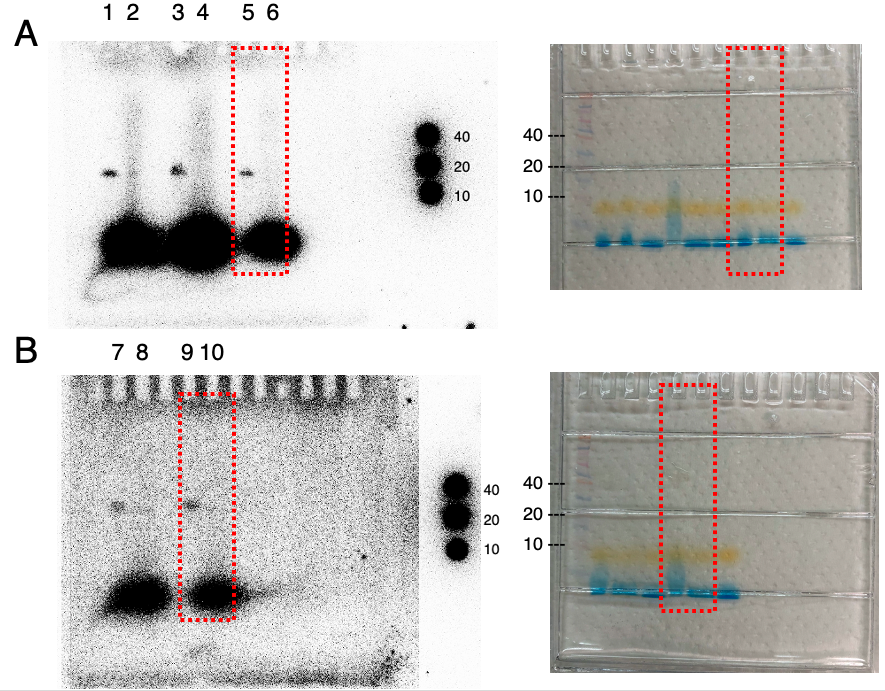


Figure S4. The gel-autoradiography image and the original gel of Figure 6.

Triplicate results (lane 1-2, lane 3-4 and lane 5-6) for [^18^F]AS69 (A) and duplicate results (lane 7-8 and lane 9-10) for [^18^F]AS69-ApoE (B) of in vivo stability analysis. Red dot squares indicate the cropped area used for Figure 6.

Table S1: Molar activity (MA), total radioactivity used and decay-corrected radiochemical yield at the start time of each radiosynthesis.

Table S2: Detailed values for *ex vivo* biodistribution analysis.
